# Linking household and health facility surveys to assess obstetric service availability, readiness and coverage: evidence from 17 low- and middle-income countries

**DOI:** 10.7189/jogh.08.010603

**Published:** 2018-06

**Authors:** Mufaro Kanyangarara, Victoria B Chou, Andreea A Creanga, Neff Walker

**Affiliations:** 1 Department of International Health, Johns Hopkins Bloomberg School of Public Health, Baltimore, Maryland, USA; 2 Department of Gynecology and Obstetrics, Johns Hopkins School of Medicine, Baltimore, Maryland, USA

## Abstract

**Background:**

Improving access and quality of obstetric service has the potential to avert preventable maternal, neonatal and stillborn deaths, yet little is known about the quality of care received. This study sought to assess obstetric service availability, readiness and coverage within and between 17 low- and middle-income countries.

**Methods:**

We linked health facility data from the Service Provision Assessments and Service Availability and Readiness Assessments, with corresponding household survey data obtained from the Demographic and Health Surveys and Multiple Indicator Cluster Surveys. Based on performance of obstetric signal functions, we defined four levels of facility emergency obstetric care (EmOC) functionality: comprehensive (CEmOC), basic (BEmOC), BEmOC-2, and low/substandard. Facility readiness was evaluated based on the direct observation of 23 essential items; facilities “ready to provide obstetric services” had ≥20 of 23 items available. Across countries, we used medians to characterize service availability and readiness, overall and by urban-rural location; analyses also adjusted for care-seeking patterns to estimate population-level coverage of obstetric services.

**Results:**

Of the 111 500 health facilities surveyed, 7545 offered obstetric services and were included in the analysis. The median percentages of facilities offering EmOC and “ready to provide obstetric services” were 19% and 10%, respectively. There were considerable urban-rural differences, with absolute differences of 19% and 29% in the availability of facilities offering EmOC and “ready to provide obstetric services”, respectively. Adjusting for care-seeking patterns, results from the linking approach indicated that among women delivering in a facility, a median of 40% delivered in facilities offering EmOC, and 28% delivered in facilities “ready to provide obstetric services”. Relatively higher coverage of facility deliveries (≥65%) and coverage of deliveries in facilities “ready to provide obstetric services” (≥30% of facility deliveries) were only found in three countries.

**Conclusions:**

The low levels of availability, readiness and coverage of obstetric services documented represent substantial missed opportunities within health systems. Global and national efforts need to prioritize upgrading EmOC functionality and improving readiness to deliver obstetric service, particularly in rural areas. The approach of linking health facility and household surveys described here could facilitate the tracking of progress towards quality obstetric care.

During the Millennium Development Goal (MDGs) era, maternal health programs in low- and middle-income countries (LMICs) prioritized increasing coverage of antenatal care and skilled birth attendant at delivery, and indeed, considerable progress was made in this regard. Between 1990 and 2014, coverage of skilled birth attendance at delivery rose from 57% to 70%, and coverage of four or more antenatal visits from 35% to 52% [1]. However, global progress to reach targets set for maternal health outcomes fell short [2]. Throughout LMICs, about 300 000 women still die every year from conditions that could be averted or addressed by medical intervention during the intrapartum period [3,4]. The increases in coverage of antenatal care and skilled birth attendance, but modest improvements in maternal health outcomes highlight the need to expand coverage in tandem with improvements in the quality of care [5,6]. High-quality obstetric care at the time of delivery has the potential to avert up to 90% of maternal deaths [7,8], 40% of intrapartum-related neonatal deaths [9], and 45% of intrapartum stillbirths [10].

In light of the ambitious Sustainable Development Goals (SDGs) to end preventable maternal, neonatal and child deaths [11], accelerated efforts are needed to improve access to and quality of care for all women and children. Tracking of trends in population coverage for maternal, newborn and child health interventions has been used to assess progress towards global goals, and hold national and international stakeholders accountable [12]. Coverage indicators express the proportion of individuals in need an intervention who receive it [13,14]. However, some indicators such as coverage of antenatal care, post-natal care and institutional deliveries reflect service contacts with the health system, and indicators of interventions received during those contact points are lacking [2]. Not all interventions along the maternal and child health continuum of care can feasibly be tracked using household surveys, the primary source of coverage data for LMICs [15-17]. In addition, household surveys cannot measure the quality with which interventions are delivered. In response to the need for valid coverage indicators reflecting both population-level receipt of interventions and the quality of intervention delivery, technical work is ongoing to develop and improve methodological approaches to coverage measurement [18]. One approach, linking care-seeking data from household surveys with service provision data from health facility assessments provides a unique opportunity to produce measures of population coverage that account for service quality, particularly during the intrapartum period [18,19].

This study aimed to gain insight into obstetric service availability, readiness and coverage in LMICs, and identify the gaps therein. Results will guide global and national efforts to improve maternal health by complementing data on coverage of facility deliveries.

## METHODS

### Data sources

Our analysis was limited to 17 countries with an available, nationally representative health facility survey conducted between 2007 and 2015, and a corresponding household survey within (+/−) two years of the facility survey. We used health facility data from the Service Provision Assessments (SPAs, n = 9) and Service Availability and Readiness Assessments (SARAs, n = 8), and household survey data from the Demographic and Health Surveys (DHS, n = 14) and Multiple Indicator Cluster Surveys (MICS, n = 3). SPAs and SARAs provide comprehensive data on the state of health systems in LMICs, with a focus on service provision. These assessments evaluate general service readiness in the dimensions: basic amenities and equipment, standard precautions, laboratory testing capacity, and essential medicines, as well as availability of specific services including obstetric care [20]. Health facilities are typically sampled from a complete listing of all health facilities in a country (master facility list), from dispensaries to tertiary hospitals, irrespective of public or non-public ownership. Generally, sampled health facilities are representative of health facilities in the formal health sector at sub-national (regional) and national levels; where resources allow, a national census of all health facilities is conducted. The core data collection tool is similar for SPAs and SARAs, and includes an inventory of equipment, diagnostics, medicines, human resources and guidelines in each facility. Complete descriptions of the survey designs, procedures and data collection tools are available elsewhere [21,22]. Our analysis was restricted to the health facilities offering obstetric services.

For estimation of population-level coverage in each country, the health facility survey (SPA or SARA) was linked to a corresponding nationally representative household survey (MICS or DHS). Both the MICS and DHS employ a two-stage cluster sampling approach, and collect information from nationally representative samples of women of reproductive age on the place of delivery for recent live births, specifically, the health facility type (eg, hospital, health center, dispensary), and managing authority (which we simplified to public and non-public) [21,22]. To reduce recall bias, we restricted our analysis to the sample of women with a recent live birth in the three years before the index household survey.

### Measures

To assess the availability of obstetric services in health facilities, four levels of functionality were defined based on reported performance of signal functions in the three months before the health facility survey. Of note, data on neonatal resuscitation were not collected as part of the two health facility surveys conducted prior to 2010 (Namibia SPA, 2009 and Rwanda SPA, 2007). Other newborn signal functions such as kangaroo mother care for low birthweight babies were excluded from the classification of functionality, as such data were not collected across all the health facility surveys included in this analysis [23]. In line with United Nations (UN) guidelines, health facilities were classified as CEmOC if they reported performing cesarean sections and blood transfusions, in addition to the seven basic signal functions: parenteral antibiotics, parenteral uterotonics, parenteral anticonvulsants, manual removal of placenta, manual removal of retained products, assisted vaginal delivery, and neonatal resuscitation [24]. Health facilities performing all seven basic signal functions were classified as BEmOC. To account for health facilities missing only one or two basic signal functions, we defined a BEmOC-2 level. All other facilities were considered “low/substandard” level in terms of availability of EmOC.

We assessed the availability of 23 items essential for obstetric and newborn care across four domains, capturing different aspects of service readiness: 1) *general requirements* including basic amenities, 2) *staff and guidelines*, 3) *equipment*, and 4) *medicines and commodities* (**Table 1**). Specifically, *general requirements* were assessed in terms of the availability of a power supply, uncontaminated water supply, sanitation facilities, communication equipment and emergency transportation. Related readiness indicators reflecting the availability of services 24/7, and the number of trained staff in a facility, previously proposed by Gabrysch and colleagues [23], were excluded due to the lack of commonly defined variables across the SPAs and SARAs. Indicators in the *staff and guidelines, equipment, and medicines* and *commodities* domains were those defined by the World Health Organization (WHO) for assessing service readiness for basic obstetric and newborn care using the SARA [25]. All 23 items across the four readiness domains were assessed by observation and direct verification. A composite measure, “ready to provide obstetric services” at the national level was defined as the percentage of health facilities with 20-23 items present.

**Table 1:** Definition of obstetric service availability and readiness

	Indicator definition
**Service availability:**	
CEmOC	Reported performance of the all 9 basic and comprehensive signal functions*
BEmOC	Reported performance of all 7 basic signal functions*
BEmOC-2	Reported performance of at least 5 basic signal functions*
Low/substandard	Performed less than 5 basic signal functions*
**Service readiness – *General requirements:***	
Power source	Reported availability of electricity for lights and communication (at a minimum) from any power source, with no break in power for more than 2 h per day during the past 7 d
Improved water source	Observed availability of an improved water source within 500meters of facility: piped, public tap, standpipe, tubewell/borehole, protected dug well, protected spring, rain water
Sanitation facilities	Reported availability of improved sanitation: flush/pour flush to piped sewer system or septic tank or pit latrine, pit latrine with slab, composting toilet
Communication equipment	Observed availability and reported functionality of a shortwave radio or phone (landline or cellular)
Emergency transportation	Reported availability and reported functionality of a vehicle with fuel that is routinely available that can be used for emergency transportation or access to a vehicle in near proximity that can be used for emergency transportation
**Service readiness – *Staff and guidelines:***	
Guidelines	Observed availability of guidelines for Integrated Management of pregnancy and childbirth (IMPAC)
Trained staff	At least one staff member providing the service trained in IMPAC in the last 2-3 years
**Service readiness – *Equipment:***	
Sterilization equipment	Observed availability and reported functionality of either a dry heat sterilizer or an autoclave
Examination light	Observed availability and reported functionality of a spotlight source (or flashlight)
Delivery pack	Observed availability of at least one delivery pack OR all the following individual equipment: cord clamp, episiotomy scissors, scissors or blade to cut cord, suture material with needle, and needle holder
Suction apparatus	Observed availability and reported functionality of suction bulb or electric suction pump or suction catheter
Manual vacuum extractor	Observed availability and reported functionality of a manual vacuum extractor
Vacuum aspirator or D&C kit	Observed availability and reported functionality of a vacuum aspirator or D&C kit
Neonatal bag and mask	Observed availability and reported functionality of a newborn bag and mask
Delivery bed	Observed availability of a delivery bed
Partograph	Observed availability of blank partographs
Gloves	Observed availability of latex gloves or equivalent
**Service readiness – *Medicines and commodities:***	
Antibiotic eye ointment	Observed availability of at least one valid unit of antibiotic eye ointment (tetracycline or other) for newborns in service area or where routinely stocked
Injectable uterotonic	Observed availability of at least one valid unit of injectable uterotonic (oxytocin or other) in service area or where routinely stocked
Injectable antibiotic	Observed availability of at least one valid unit of broad-spectrum injectable antibiotic (gentamicin, penicillin, or ampicillin or ceftriaxone) in service area or where routinely stocked
Magnesium sulphate	Observed availability of at least one valid unit of injectable magnesium sulphate or diazepam in service area or where routinely stocked
Skin disinfectant	Observed availability of skin disinfectant in service area or where routinely stocked
IV solution with infusion set	Observed availability of infusion set and intravenous fluids (normal saline or Ringers Lactate or Dextrose 5%)

*Basic signal functions: parenteral antibiotics, parenteral uterotonics, parenteral anticonvulsants, manual removal of placenta, manual removal of retained products, assisted vaginal delivery, and neonatal resuscitation. Comprehensive signal functions: cesarean section and blood transfusion.

### Statistical analysis

Descriptive analyses were conducted to assess obstetric service availability and readiness at health facilities, and the proportion of facility deliveries. We reported the percentage of health facilities in each country meeting respective indicator criteria, and the median level across all countries included in our analysis. Medians were not weighted by country-level population size or the number of facilities offering obstetric services. Results were disaggregated by urban-rural location based on country specific definitions. For each country and indicator, urban-rural inequalities were derived by calculating the absolute differences in indicator values between urban and rural areas. Information on the urban or rural location of health facilities was not available in health facility survey data sets for Kenya, Namibia, Nepal and Rwanda.

We linked health facility and household survey data at the aggregate level to estimate the proportion of deliveries occurring in different service environments. We defined strata of health facilities based on facility type (hospital, health center, and dispensary) and managing authority (public, non-public). We then estimated indicators of service availability and readiness for each stratum, using the health facility survey data. To estimate population-level coverage indicators, we estimated the proportion of recent live births occurring in a stratum from the household survey, and then multiplied these proportions by service availability and readiness indicators for that stratum. This approach assumed that all the women who delivered in a health facility assigned to a specific stratum experienced the “average” service availability and readiness for that stratum. Linking health facility and household survey data allowed us to consider gaps in population level coverage of obstetric services, overall and separately by urban and rural areas. To show coverage and quality gaps, we plotted the proportion of deliveries in facilities “ready to provide obstetric services” by coverage of facility deliveries.

All analyses were conducted using STATA 14.2 (College Station, TX, USA) and accounted for the complex survey sampling of each survey.

## RESULTS

The majority of countries meeting our eligibility criteria (14/17) were in sub-Saharan Africa, with three countries (Bangladesh, Haiti and Nepal) outside the region (**Table 2**). A total of 11 500 health facilities were assessed in the 17 health facility surveys, of which 7545 (66%) reported offering obstetric services, and were included in the analysis. The number of sampled health facilities offering obstetric services ranged from 89 in Togo to 1273 in Democratic Republic of Congo (DRC). The total number of recent live births in the corresponding household surveys was 103 983, ranging between 2086 live births in Nepal and 11 279 in the DRC.

**Table 2:** Description of the health facility and household surveys included in the analysis

	Health facility surveys				Household surveys			
	**Type**	**Year**	**Number of facilities sampled**	**Number of facilities offering obstetric services**	**Type**	**Year**	**Number of live-births reported**	**Facility deliveries (%)**
Bangladesh	SPA	2014	1596	586	DHS	2014	4492	37
Benin	SARA	2013	189	137	DHS	2011/12	9111	87
Burkina Faso	SARA	2012	686	604	DHS	2010	10 364	66
DRC	SARA	2014	1555	1,273	DHS	2013/14	11 279	80
Haiti	SPA	2013	907	395	DHS	2012	5414	36
Kenya	SPA	2010	695	403	DHS	2008/09	4082	43
Malawi*	SPA	2013/14	977	540	MICS	2013/14	7576	89
Mauritania	SARA	2013	232	126	MICS	2011	3629	65
Namibia*	SPA	2009	411	256	DHS	2006/07	4020	81
Nepal	SPA	2015	992	623	MICS	2014	2086	55
Rwanda*	SPA	2007	538	404	DHS	2007/08	3568	45
Senegal	SPA	2015	483	362	DHS	2015	8954	78
Sierra Leone	SARA	2013	455	420	DHS	2013	8524	54
Tanzania	SPA	2014/15	1200	951	DHS	2015/16	7050	64
Togo	SARA	2012	100	89	DHS	2013/14	5012	73
Uganda	SARA	2013	209	126	DHS	2011	4909	57
Zimbabwe	SARA	2014	275	250	DHS	2015	3913	79
**Total**	–	-	11 500	7545	–	–	103 985	–

DRC – Democratic Republic of Congo, DHS – Demographic and Health surveys, MICS – Multiple Indicator Cluster Surveys, SPA – Service Provision Assessment, SARA – Service Availability and Readiness Assessment, TZ – Tanzania

*Countries with facility censuses.

### Obstetric service availability in health facilities

**Figure 1:**
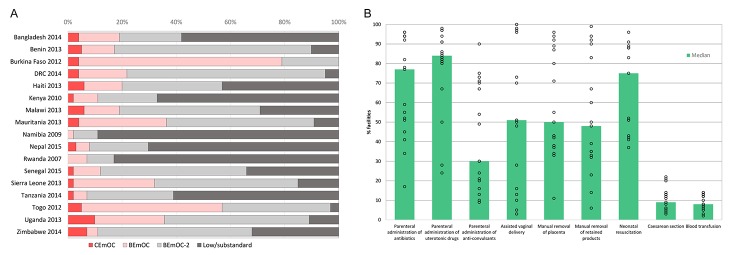
Obstetric service availability in health facilities in 17 low- and middle-income countries, 2007-2015. **A**. Percentage of health facilities by emergency obstetric care capability. **B**. Percentage of health facilities by availability of obstetric signal functions.

Overall, the national-level capacity to provide EmOC was inadequate, yet some countries fared better than others (**Figure 1**). Based on the performance of obstetric signal functions, the across-country median percentage of CEmOC facilities was 4% (range 0-10%), and the median percentage of facilities with at least full BEmOC capability was 19% (range 2-79%) (panel A in **Figure 1**). Analysis of the performance of individual signal functions indicated variations by signal function and country (panel B in **Figure 1**). Parenteral administration of uterotonic drugs (median 84%), and antibiotics (median 77%) were the most frequently performed signal functions in three months before the health facility survey, followed by neonatal resuscitation (median 68%), assisted vaginal delivery (median 51%) and manual removal of placenta (median 50%). The widest range of country performance of obstetric signal functions was for assisted vaginal delivery. The majority (>95%) of facilities in Burkina Faso, DRC, Mauritania, Senegal and Togo reported performing an assisted vaginal delivery during the three months before the facility assessment, yet performance of this signal function was rare (<5%) in Kenya and Namibia. The two comprehensive obstetric signal functions, blood transfusion and cesarean section, were provided at least once in about one in ten facilities offering obstetric services (median 8% and 9% respectively).

### Obstetric service readiness in health facilities

**Figure 2:**
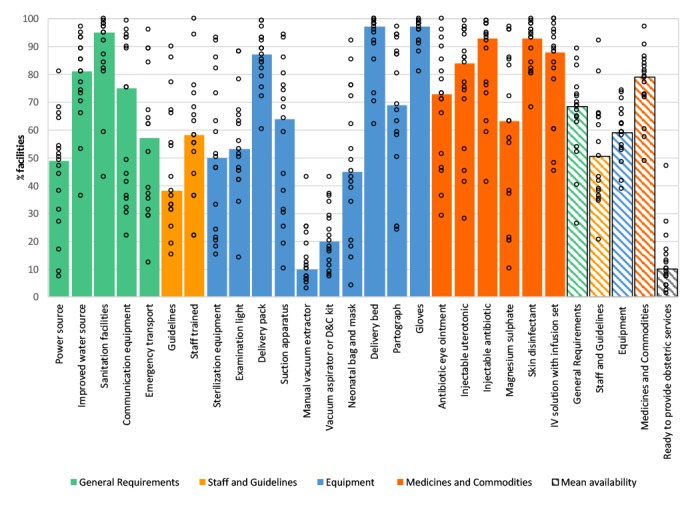
Obstetric service readiness in health facilities in 17 low and middle-income countries, 2007-2015.

Overall, the availability of *general requirements, staff and guidelines, equipment* and *medicines and commodities* to support the delivery of childbirth services was sub-optimal (**Figure 2**). A median of 95% of facilities across countries had a functioning sanitation facility (range: 43-100%), 81% had an improved water source (range: 36-97%), and 49% had a reliable power supply (7-81%). The ability to facilitate the timely referral of obstetric emergencies was poor. While three-quarters of facilities had a working telephone or shortwave radio (median 75%, range: 22-99%), just over half had emergency transport for the referral of obstetric emergencies (median 57%, range: 12-96%). A median of 58% of health facilities had at least one health worker who had received in-service training in any aspect of essential childbirth in the previous 1-3 years (range: 22-100%). Guidelines on essential childbirth were documented in about 38% of health facilities (range: 15-90%).

Basic *equipment* for safe delivery, such as gloves, delivery beds, and delivery packs were almost universally available at the time of the health facility assessments (median 97%, 97% and 87% respectively) (**Figure 2**). Sterilization equipment, examination lights, suction apparatus and partographs were somewhat less available (median 50%, 53%, 64% and 69%, respectively). Equipment and supplies essential for removal of retained products, assisted vaginal delivery and neonatal resuscitation were in short supply. Median availability of a manual vacuum extractor, and vacuum aspirator or D&C kit was 10% (range: 3-43%) and 20% (range: 7-43%) of health facilities, respectively. In terms of essential *medicines and commodities*, injectable antibiotics (median 93%, range: 41-99%), injectable uterotonics (median 84%, range: 28% - 99%) and skin disinfectants (median 93%, range: 68-100%) were in good supply. Compared to the other medicines and commodities assessed, injectable magnesium sulphate had the lowest availability (median 63%), and was more variable across countries (range: 10–97%).

**Figure 3:**
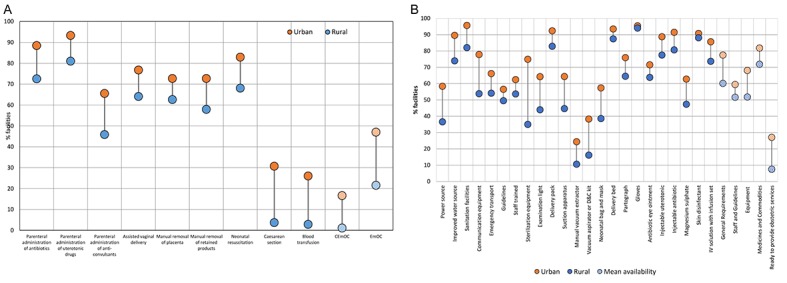
Urban-rural inequalities in obstetric service availability and readiness in 17 low and middle-income countries, 2007-2015. **A**. Performance of obstetric signal functions by urban-rural location. **B**. Availability of general requirements, staff and guidelines, equipment and medicines and commodities by urban-rural location.

Composite measures of health facility readiness indicated that the domain with the highest median availability was *medicines and commodities* (79%), followed by *general requirement*s (68%), *equipment* (59%), and *staff and guidelines* (51%) (panel A in **Figure 3**). The median percentage of facilities of “ready to provide obstetric services” (ie, ≥20 of 23 items) was 10%, ranging from 1% in the DRC to 47% in Malawi.

### Urban-rural disparities in obstetric service availability and readiness in health facilities

National-level measures of service availability and readiness masked differences among facilities in urban and rural areas (**Figure 3**). Facilities in rural areas performed fewer signal functions than those in urban areas, with gaps exceeding 20 percentage points in the availability of parenteral administration of anti-convulsants, cesarean section and blood transfusion (panel A in **Figure**). In contrast, across countries, the urban-rural gap was narrowest for manual removal of placenta, performed in a median of 73% of urban and 63% of rural facilities. A median of 43% of facilities in urban areas were designated as EmOC vs 14% of facilities in rural areas, an absolute difference of 29 percentage points. We also found urban-rural disparities in all four domains of facility readiness assessed (panel B in **Figure 3**). For instance, the median national availability of sterilization equipment was 51%, but the median availability was 75% in urban and 35% in rural facilities, representing an absolute difference of 40 percentage points. Across the 13 countries with relevant data, the median percentage of facilities “ready to provide obstetric services” was 8% in rural areas compared to 27% in urban areas.

### Coverage of obstetric services for facility deliveries

**Figure 4:**
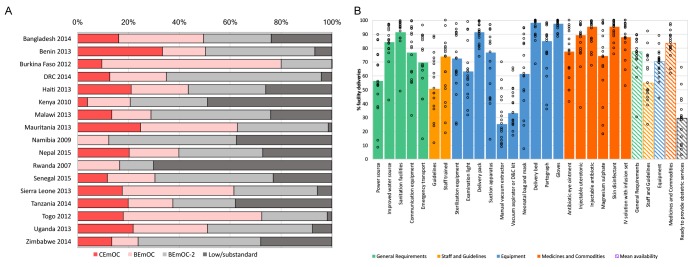
Coverage of obstetric services among women delivering in health facilities in 17 low- and middle-income countries, 2007-2015. **A**. Percentage of facility deliveries by emergency obstetric care capability. **B**. Percentage of facility deliveries by obstetric service readiness.

Coverage of facility deliveries varied between 36% in Haiti and 89% in Malawi, with a median of 65% across all countries (**Table 2**). Linking household and health facility surveys, we estimated population-level coverage of obstetric services. Overall, patterns in the population level coverage of obstetric services were similar to those observed with facility level service availability and readiness indicators (**Figure 4**). However, results from the linking approach did show a somewhat better situation. Notably, a median of 28 and 30% of facility deliveries occurred in facilities with blood transfusion and cesarean section capabilities, compared to a median of 8 and 9% of facilities with such capacity (**Figure 4**). Among women who delivered in a facility, a median of 42% took place in facility with EmOC (basic or comprehensive) functionality, with a low of 12% in Namibia and a high of 80% in Burkina Faso. The median percentage of facility deliveries in facilities “ready to provide obstetric services” was 29%, with a high of 66% in Nepal (**Figure 4**).

**Figure 5:**
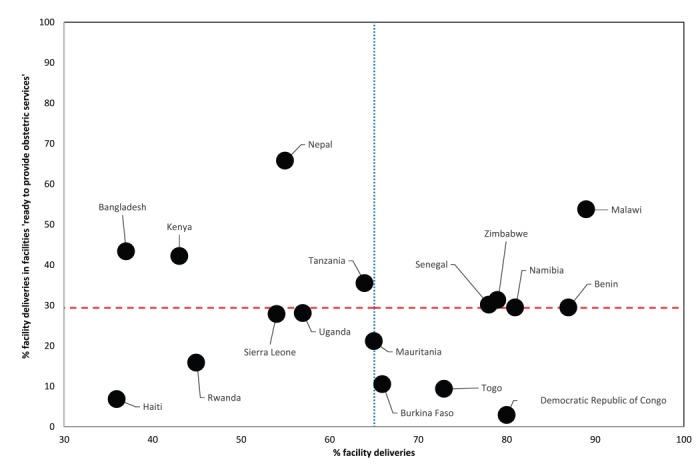
Obstetric service readiness by coverage of facility deliveries in 17 low and middle-income countries, 2007-2015.

Countries similar in terms of coverage of facility deliveries showed different degrees of obstetric service readiness (**Figure 5**). The median coverage of facility deliveries (65%) and the median obstetric service readiness (29%) were used to compare countries. Of the nine countries with greater than median (≥65%) coverage of facility deliveries, only Benin, Malawi, Namibia, Senegal and Zimbabwe had more than 29% of those take place in facilities rated as “ready to provide obstetric services”. Despite the below median coverage (<65%) of facility deliveries in Bangladesh, Kenya, Nepal and Tanzania, the proportion of facility deliveries in facilities designated as “ready to provide obstetric services” was between 36% and 66%, well above the median of 29%. Countries like Haiti and Rwanda had matching low coverage of facility deliveries (<65%) and obstetric service readiness (<29%).

## DISCUSSION

Our analysis documented low levels of obstetric service availability, readiness and coverage in 17 LMICs, with variation between and within countries. Across countries, among women delivering in a health facility, a median of 42% and 29% occurred in facilities classified as EmOC and “ready to provide obstetric services”, respectively. By contrast, a median of 10% and 19% of facilities were classified as EmOC and “ready to provide obstetric services”, respectively. In terms of the reported performance of signal functions at health facilities, assisted vaginal delivery and parenteral administration of anticonvulsants were the least performed basic signal functions. The former is not surprising given similar observations in other multi-country studies in LMICs [26-28], and the relatively lower availability of manual vacuum extractors documented in the present study. The low performance of parenteral administration of anti-convulsants and corresponding low supply of injectable magnesium sulphate suggests supply chain issues. To reduce maternal mortality attributable to pre/eclampsia, anticonvulsants should be readily available, along with provider training on administration. Yet, *staff and guidelines* represented the service readiness domain with the lowest availability across the 17 countries. We found important training deficits even with the relaxed criteria that at least one health provider was trained in any aspect of essential childbirth care in the past one to three years. There is an imperative need to train health workers and improve performance.

Our results have several broader implications for the improvement of obstetric services in LMICs. First, our study challenges the prioritization of increasing coverage of institutional deliveries with little consideration of the quality of service delivery. While increasing population coverage of facility delivery is an important vehicle for increasing access to interventions, it is not appropriate to assume that once contact with the health system is made, the appropriate care will be received [29]. Without complementary data on the quality of service delivery, globally tracked indicators such as coverage of facility deliveries will overestimate achievements towards improving maternal health. Our results indicate that higher demand for and access to institutional delivery was not always accompanied by an adequate level of facility readiness to provide obstetric services ie, “good quality”. Above median levels of obstetric service readiness and coverage of facility deliveries were found in only five countries (Benin, Malawi, Namibia, Senegal and Zimbabwe). The remaining countries either had achieved 1) high coverage for facility births that was not matched by high obstetric service readiness (eg, DRC and Togo), or 2) high obstetric service readiness with only a minority of births occurring in facilities (eg, Nepal and Bangladesh), or 3) had similarly low coverage of facility deliveries and obstetric service readiness (eg, Haiti and Rwanda). These findings lend support to the need for a double-pronged approach focused on expanding access and improving quality of obstetric services.

Second, while we expected to find systematic urban-rural disparities in the obstetric service availability and readiness, the magnitude of the gaps indicate the need for substantial political will to increase resource investments in rural areas. It is worth noting that urban facilities were more likely to be designated as EmOC and “ready to provide obstetric services”. Women in urban settings benefit from the dual advantage of the greater access to and quality of care. Given large segments of the population in LMICs live in rural areas and the disproportionately poorer access to and quality of obstetric services in rural areas, the development of sustainable health systems in rural settings is imperative.

Third, our results indicate the need to prioritize resource allocation to facilities serving a larger proportion of pregnant women. While a median of 10-19% of facilities were classified as “ready to provide obstetric services” or EmOC, results from the linking approach showed the median percentage of institutional deliveries occurring in facilities classified as “ready to provide obstetric services” or EmOC was 28% and 42% respectively. A possible explanation of this pattern is that women bypass poor functioning facilities, electing to deliver in facilities with better obstetric service readiness [30]. Alternatively, facilities serving more women may indeed offer better obstetric service readiness. Previous work has demonstrated an association between the volume of deliveries and quality of maternal care, with lower quality of care in facilities with low volumes [31]. In many LMICs, lower level facilities such as birthing centers or maternities were designed as a strategy to handle uncomplicated deliveries [32]. These facilities are unlikely to provide EmOC, but with access to emergency transportation and communication means, they can facilitate the referral of women with obstetric complications to higher level facilities capable of offering EmOC. Prioritizing the expansion and improvement of high delivery volume facilities over strengthening lower level facilities will in part depend on the specific country-context, geography, population density, service demand, decentralization, referral systems and funding and resource constraints. On the other hand, in light of the urban-rural disparities identified, facilities in rural areas should be prioritized for improvements in infrastructure, human resources, equipment, and drug and commodity supply chains, along with strategies to promote the use of obstetric services, and improved referral to EmOC. The private sector is expected to play an increasing role in offering obstetric services in some LMICs [33], especially given current efforts to incentivize institutional deliveries by employing various financing schemes [34].

Fourth, there is an imperative need to generate a consensus on standard indicators, harmonize data collection tools and develop innovative methodologies to monitor the quality of maternal, neonatal and child health interventions across countries and over time. The service readiness domains and indicators considered do not capture all aspects of quality of care (eg, respectful care, client satisfaction, provider competence, and adherence to standards of obstetric practice). An enabling environment with functioning equipment, adequate drugs and competent staff is a prerequisite but not a guarantee of receipt of good quality care by those in need [13]. To comprehensively assess the adequacy of obstetric care, health facility assessments such as the SPA and SARA need to collect details on staffing credentials and the availability of trained staff. The limited availability of such data in national health facility surveys and the lack of standard indicator definitions across health facility surveys restricted the scope of quality constructs that could be assessed and the geographic representation of the present study. The rapid expansion and increased frequency of national health facility assessments will allow the tracking of global and national trends in the quality of service provision. Additionally, efforts such as the Improving Coverage Measurement project will help guide the global community generate a consensus on a set of core indicators reflecting the content of care, associated measurement tools, and standard methodologies to improve coverage measurement.

Our study is not without limitations. While this study sheds light on obstetric service provision across 17 LMICs, the countries in our analysis are not representative of all LMICs, and sampled facilities assessed may not be representative of all service providers in a country. Notably, in countries actively promoting home-based models of care such as skilled birth attendance for homebirths, the results presented here may underestimate coverage of obstetric services. Sampling of all service providers rather than health facilities will allow for a complete understanding of obstetric service provision than a sample of health facilities, and provide more reliable estimates of coverage. Further research is needed to determine the validity and feasibility of this sampling approach. Lastly, the data sources and methodology used are prone to several limitations including the time difference between the household and health facility surveys and various forms of bias [19,35]. Of note, while we linked health facility and household survey data at an aggregate level, which is one of the proposed linkage methods for independently sampled surveys, there have been no validation studies of this method [19,36,37].

## CONCLUSIONS

Despite these limitations, we used the most recent and best available nationally-representative health facility and household survey data to generate population-based estimates of the coverage of obstetric services within (urban-rural) and between countries. Our analysis adds to the growing evidence on the coverage and quality of maternal health services in LMICs [5,26,31,38]. The proposed data driven prioritization of improvements in both obstetric service utilization and quality of services will lead to better maternal and neonatal health outcomes. The increased emphasis on evaluating the quality of care will most likely raise the prominence of health facility assessments, and urge the increasing development of innovative methodologies to measure quality and to generate quality-adjusted measures of population coverage in the global health sector.
